# Medical Association Journals and the Postal Laws

**Published:** 1910-03

**Authors:** 


### EDITORIAL DEPARTMENT.

### MEDICAL ASSOCIATION JOURNALS AND THE POSTAL LAWS.

**Thirteen Hundred Tons of Free Medical Journals Sent Annually Through the Mails at Second-class Rates, in Violation of the Law! An Important Factor in That $17,000,000 Deficit.**

It is not generally known that the *Journal of the American Medical Association* and most of the State Medical Association journals, including Texas, are, and for many years have been, violating the postal laws in two essential particulars; but such is a fact. In an official letter from the Acting Third Assistant Postmaster General addressed to a party in South Carolina in reply to inquiries, a copy of which letter is before me, he holds that members of an association who receive the association's journal in consideration of the payment of membership fees or annual dues are not *bona fide* subscribers, as prescribed by the department as a condition precedent to the privilege of second-class mailing rates, and that such journals shall not carry advertisements other than those that relate strictly to the affairs of the association and the purposes of such organization.

The second-class mailing privilege (one cent per pound) having been obtained by the representation that these medical publications have a large *bona fide* subscription list, this savors of misrepresentation and (unintentional) fraud, and it may constitute grounds for action at law, and certainly, warrant for the withdrawal of the privilege of second-class mail. A subscriber is one who orders and pays for a journal,—at his discretion,—and not a member who has it sent to him in consideration of the payment of dues.

This is a very serious matter, and I call the attention of our Trustees to it. The *Journal of the American Medical Association* has caught on—or, been caught up with—and is hedging, as I pointed out in last issue, by separating the subscription to the journal from the membership fee; the latter being now announced as $1 a year, and the subscription to the journal—discretionary—at $4. This must be done by our own Association at the Dallas meeting in May, and I shall introduce a resolution to that effect.

The American Medical Association journal has also been brought to see that all these years it has been carrying advertisements not allowed by the postal laws—automobile ads., and such like, and has doubtless caused to be introduced in Congress a bill to legalize the carrying of such advertisements. At any rate, Mr. Dodds has introduced such a bill (House Bill 17543, by Dodds, January 10, 1910), and the *Journal of the American Medical Association* doubtless inspired it. The cap that fits the "Octopus" fits also its tentacles—the State Association journals.

The Acting Third Assistant Postmaster General, after quoting in full the postal laws bearing upon the subject and giving the opinion of the Attorney General of the United States in support of his position and construction, comments as follows:

*"In all cases it must appear that it is left to the option of the member whether he will subscribe or not. The subscription must not be forced or made obligatory upon him.*

"This law unquestionably contemplates that there shall be as a prerequisite to such entry a public demand for the publication on its merits evidenced by a list of subscribers who desire it and pay for it.

"Many fraternal orders and other similar organizations provide by resolution or by-law that a certain portion of each member's dues, or per capita tax, etc., shall be set aside for the payment of his subscription to some paper which is published by the society or adopted as its official organ.

"At first sight it might appear that a list of such members should be recognized as a 'legitimate list of subscribers,' but upon a careful consideration of this method of securing a subscription list, it is found to be a meeting of the requirements of the act in form rather than in substance, as it is usually the fact that the price of membership, or the amount of dues payable is the same, whether the members desire the publication or not; that is to say, it is not left to the option of a member to receive the publication or not, as he sees fit, and to have his dues increased or decreased accordingly.

"In the last analysis, therefore, the publication is virtually thrown in free as a concomitant of membership, the expense of publishing and circulating the paper merely being paid out of dues accruing from membership in the society. The effect of all this is that the members, being the society, publish a paper and furnish it to themselves free. The society is therefore in reality both the publisher and subscriber. Viewed in this light not only does the paper not have a 'legitimate list of subscribers,' but is literally circulated free, and, therefore, *comes clearly within the prohibition of the statute.*

"The Department has uniformly held that publications containing advertisements in the interests of other persons or concerns, than the society or trades union or institution of learning which the paper represents, are not entitled to the privileges of that Act.

"Considering, therefore, that Congress undoubtedly intended to extend the second-class mailing privileges to certain publications under the conditions above set forth, it is clear that if these publications are to enjoy the second-class mailing privileges under an Act other than the one specially designed for them, the requirements of the alternative Act should be fully met. They certainly can not enjoy the privileges of both acts without the restrictions of either. In this connection two observations may be made:

"1. It is a fact disclosed by answers to many inquiries that but few of the organizations whose publications are entered under the Act of March 3, 1879, and having the 'list of subscribers' based on a by-law or resolution providing for setting aside a portion of the members' dues, are actually abiding by the resolution, but, on the contrary, the monies paid on account of alleged subscriptions enter into the general funds of the organization and the cost of printing and circulating the publication is merely paid therefrom.

"2. It is manifestly an injustice to publishers competing in the same or a similar field and having papers which would be patronized by the members of an organization to be compelled to secure and maintain a list of subscribers based on the drawing power of the paper as such, whereas the competing organization papers' list of subscribers is composed of members who were drafted in under a by-law of the organization and receive the paper as one of the benefits of membership, and not on account of voluntary subscription. Publishers of other papers have entered complaints asserting that people will not pay for that which they can receive free. Consequently, those receiving a paper through the medium of membership in a society are not disposed to pay for some other paper in the same field."

President Taft calls the attention of Congress to the statement of the Postmaster General that the second-class mail falls short $17,500,000 of paying expenses. The Third Assistant Postmaster General recommends very drastic measures to be applied to the small medical and other journals, such as limiting sample copies to 10 per cent of issue, and refusing mail to copies sent to all subscribers who do not *renew and prepay* their subscriptions within four months after expiration of same. And yet he permits tons upon tons of medical journals to be sent at second-class rates, in violation of the law as interpreted by himself backed by the opinion of the Attorney General of the United States. For twenty-five years and more the *Journal of the American Medical Association* has been sending copies to its members in consideration of payment of their dues, and carrying advertisements outlawed by said opinion. In twenty-three States the Medical Association issues a monthly journal which is sent free to its members—in violation of the law as pointed out above. *The Journal of the American Medical Association* sends 50,000 copies each week, averaging, say, half pound. That is, 52 weeks in a year, equals 2,600,000 copies, or six hundred and fifty tons of mail that should pay postage. It is safe to estimate the output of the twenty-three State journals at about the same. This would make *thirteen hundred tons a year.* The Dodds bill, if passed, would legalize and make permanent this burden on the mail,—as to the advertisements. The Third Assistant Postmaster General has, in effect, put up the bars against the pigs, and left ten panels of fence down for the hogs and cattle. To give the State and National journals such advantage over the journals having a paid subscription list is a discrimination ruinous to the smaller publications, for as the Postmaster General says, "a man will not pay for that which he gets free." It is tantamount to *a combination in restraint of trade,* inasmuch as it creates a monopoly for the favored journals, and destroys competition and destroys the independent journals. The TEXAS MEDICAL JOURNAL, now in the twenty-fifth year of sustained patronage and usefulness, protests against such an unjust discrimination.

[Exchanges will please copy.—DANIEL.]

---

LET IT BE REMEMBERED THAT at the May meeting of the Texas State Medical Association at Dallas the election of an editor of *The Texas State Journal of Medicine* and Secretary of the State Medical Association will take place. We must select an able and popular man. The rank and file, and not alone the House of Delegates, must have a voice in the matter. No one connected with any other journal should be thought of for the office.

That a proposed amendment to the constitution will come up, limiting membership in the Association to white persons of the regular profession, except such worthy members of the several pathies as will drop their sectarian titles. The rank and file must have a vote here, as provided for in the constitution (referendum), but which provision has always heretofore been ignored.

That I shall introduce and advocate a resolution instructing the Trustees of the *Journal* to have all papers read at meetings (subject to approval of the Publishing Committee) bound in one volume and sent free to every member. The reasons for this are obvious. It is impossible to publish these papers in the *Journal* simultaneously, and members complain of discrimination. Some papers do not see daylight in twelve months. The cost will be comparatively insignificant; that of the "Transactions" under the old regime being only 77 cents per volume, postpaid, each volume in cloth and containing 650 pages. No one has the *Journal* bound. The members care only for the papers and discussions. The *Journal* can still be issued as the Association newspaper, and sent to members who care for the lists of members of societies and the feeble and partisan editorials, and who are willing to pay for it. The rank and file should and must have a vote on this.

That I will introduce a resolution that the annual dues shall be $1.00, and the subscription to the *Journal* $1.00, and that the latter shall be discretionary with the members. In this connection read the leading editorial in this issue. By the bye, parenthetically, this matter will be brought to the attention of the Postmaster General, the Congressional Committees on Postoffices and Mail Routes, and doubtless laid before the Senate. The reasons for this are obvious. Every member of every medical society must pay $2.00 a year, half of which is supposed to be the subscription price of the *Journal*, but it is not. If one does not want the *Journal* he has to pay for it all the same, and there are many who do not need it, e. g., Dr. Cantrell and his brother Will have each to pay for a copy; Drs. Becton & Becton, Drs. Saunders & Son, Drs. Osborn & Son—all firms—have to pay for a copy for each member; every individual member of an editorial staff has to pay for a copy; every individual member of the staff of every State asylum or State or private sanitarium; every individual member of the Faculty of the University Medical College; every individual physician connected with the Health Department has to pay for a copy, when one for each body would be sufficient. In large office buildings a number of doctors club, each taking a certain line of journals and exchanging. Every one in the club (supposing him to be a member, and if he is not Chase and Simmons say he is a scab and not in good standing) is forced to take and pay for this tentacle. Is there either sense or justice in this? The rank and file shall have a voice in this: a constitutional right, and by the Eternal it shall be exercised.

